# Being well together? Promoting health and well-being through more than human collaboration and companionship

**DOI:** 10.1136/medhum-2018-011601

**Published:** 2019-02-28

**Authors:** Robert G W Kirk, Neil Pemberton, Tom Quick

**Affiliations:** Centre for the History of Science Technology and Medicine, University of Manchester, Manchester, UK

**Keywords:** medical humanities, mental health care, veterinarian, cultural studies, cultural history

## Abstract

Being well together, an inaugural Research Forum, will critically examine the myriad ways humans have formed partnerships with non-human species to improve health across time and place. Across the humanities and social sciences, a growing body of scholarship has begun to rethink the prominence of the ‘human’ in our accounts of the world by exploring the category less as an individualised essence and more as a temporal process of becoming. From this perspective, being human becomes a process of ‘becoming with’, performed through interactions with non-human others. This paper introduces a diverse collection of studies, originally presented at a workshop held at the University of Manchester in 2018, which explored how emergent approaches within animal studies might productively and playfully engage with the medical humanities. In each case, human health and well-being is shown to rest on the cultivation of relationships with other species. Being well is rethought and remapped as a more than human process of being well together. Collectively, this research forum invites reflection on what the medical humanities might look like from a more than human perspective.

The humanities and social sciences have begun to rethink the prominence of the ‘human’ in our accounts of the world by exploring the category less as an individualised essence and more as a temporal process of becoming. The human as a process, crucially, is never undertaken alone.1 Rather, being human is a process of becoming *with*, operating through interactions with others, including non-human others.2 A growing body of interdisciplinary scholarship, loosely collectivised under the rubric ‘animal studies’, seeks to understand how human–animal relationships have shaped cultures, politics, societies, identities and ways of life.3 As an academic field, animal studies shares many characteristics with medical humanities. Not least interdisciplinarity, a commitment to making a difference in the world, and an opendebate as to its purpose, identity and name.4 Both fields share an interest in living well. Within animal studies, a prominent theme is to understand how to live with other species so that all prosper within a mutually beneficial, although not necessarily equal, collective (often expressed through the language of ‘flourishing’).5 This concern resonates closely with that primary object of the medical humanities: health and well-being. Both share an interest in what might be thought of as ‘being well’.

Yet, conversations across the two fields are few. A prominent difficulty is the productive relationship between the medical humanities and disability studies on the one hand, and the tension between animal studies and disability on the other (which we return to below). While acknowledging the very real challenges in aligning these fields, we propose that concepts and approaches emerging from animal studies may have real value when applied to medicine, health and well-being. Put succinctly, if being human is a process of becoming together, by extension being well becomes a process of being well together. This opening conceit was explored at a workshop convened by the authors at the University of Manchester and supported by the Wellcome Trust (UK). Over 3 days, participants discussed how animal studies and medical humanities might be allied to better understand health as an outcome of human interactions with non-human forms of life. This inaugural Research Forum presents papers which critically examine the myriad ways humans have formed partnerships with non-human species to improve health across time and place. In the spirit of a Research Forum, each contribution presents an initial and preliminary sketch. A proposition intended to provoke dialogue and encourage critical reflection on what it may mean to reframe the medical humanities as a field concerned with more than human flourishing. We are, after all, entangled in a shared process of becoming. It would be careless not to welcome your response.

## Health and the human–animal relationship

The late 20th century witnessed the recognition, growth and diversification of the human–animal relationship as a means to promote health and well-being. Clinical examples include the use of maggots to treat chronic wounds and the post-surgical use of leeches to aid healing.6 In wider society we might consider service animals, such as guide dogs, diabetes alert dogs and emotional support animals. In the home, pets are increasingly recognised as contributors to emotional well-being, with companion animals particularly important to those who are otherwise at risk of social isolation.7 Expanded to include concepts such as the ‘human’ microbiome in the opening decades of the 21st century, these entanglements may be tentatively thought of as forms of ‘multispecies medicine’. In each case, human health and well-being rests on the cultivation of relationships with other species. Being well is rethought and remapped as a process of being well together.

The proposition that human health is inseparable from animal health has a long history.8 Nevertheless, within modern medicine, consideration of the non-human animal remains relatively marginal. Their relevance to health and well-being obscured. Within the biomedical sciences, the interconnection of human and animal health is central to experimental research although frequently veiled from public attention due to the contentious nature of animal based science.9 In clinical medicine and wider society, the role of animals in promoting health is all too often located on the hinterland where ‘mainstream’ medicine ends and ‘alternative’ therapies begin. Indeed, one of the key challenges in taking seriously the proposition that human relations with animals have real consequence for health and well-being is a perceived association of these practices with fringe or complementary medicine.

One important exception is zoonosis. Here the human–animal relationship appears as a prominent concern. In recent years, avian influenza, swine influenza and Ebola, among other zoonotic outbreaks, have refocused attention on the inseparability of human and animal health. The global nature of emergent and new zoonotic diseases have empowered calls for a coming together of human and veterinary medicine under the banner of ‘one health’ and ‘one medicine’ (calls which in themselves are not new but are experiencing renewed credibility).10 Such movements operate within an ethical regime which considers animal welfare predominantly as a factor in promoting and securing human health. Moreover, while ‘one health’ engages with the integrated and ‘messy’ relationship of human and animal health, it does so very much within the biomedical logic of control and eradication of disease. It recognises the integral importance of the messiness of human–animal relations for health, but does so only to clean up the mess. Without prejudicing the focus of one medicine on zoonotic disease, theoretical approaches within animal studies move in a different direction in their attempt to embrace and preserve complexity while flattening any assumed hierarchy of value across species.

Accordingly, in this *Research Forum*, we explore how medicine has harnessed human–animal interaction to promote health and well-being. In doing so, we investigate how interactions with animals have been understood to bring positive benefits to humans (characterised as ‘zooeyia’) but also to the animals themselves.11 Concern for the animal in a given partnership quickly comes to fore, although in highly situated ways, once they are included within therapeutic settings. Unsurprisingly, human obligations toward animals aremore prominent with some species than others. At the end of their working lives, for instance, the contemporary guide dog is ‘retired’ in the form of rehoming to take up a new life as a pet. This practice both recognises their labour and values their life independent of the work. In contrast, the retirement of a medical maggot is quite different. Maggots are disposed of having been designated clinical waste. Both species come into intimate relationships with the human. Yet, for one it leads to enhanced value whereas for the other it leads to perceived contamination necessitating their careful destruction.

Counterpoising such diverse practices can, therefore, reveal the embedded cultural assumptions which frame the way we value non-human species. It also challenges such assumptions. A prevailing healthcare concern, for instance, is that patients will resist the application of maggots due to their association with dirt, disease and death. One response to this has been technological: maggots are supplied within so-called ‘bio-bags’ to be placed on wounds. The bio-bag responds to perceived cultural concerns by circumventing the need to place animals directly within a wound. Safely packaged in a sterile 'bag' the he risk of maggots migrating from the wound is removed and the maggots themselves obscured. However, in practice, anecdotal reports suggest patients respond to larval treatment relatively enthusiastically and often express gratitude toward their non-human healers. Ethnographic study of larval therapy may, therefore, prove to a rich area for understanding how societal and cultural values shape and are shaped by health interventions as well as how the same utilise and transform how we value relations with animals.

We recognise that this is a research topic in search of a name, in part as its diverse examples challenge and transcend humanistic categories. For the purposes of the workshop we tentatively adopted the term ‘multispecies medicine’ to capture the varied ways in which interactions with non-human life have been harnessed within a process of being well together. We began with a question: How have non-human forms of life contributed to enhancing human health and well-being? We proposed that investigating how non-human life has contributed to promoting human health and well-being may invite a reframing of biomedicine as a more than human enterprise. While diverse, the varied ways in which human–animal interaction has been mobilised within medicine philosophically share an ecological vision of health focused on collectives as opposed to isolated individuals. Health and well-being become products of relations between forms of life and their shared physical–social environment. Studying this process reveals how medicine has shaped, and been shaped by, changing human–animal relations in society. Investigating how medicine has been performed across species boundaries will reveal how historically situated cultural perceptions of a species shaped experiences and acceptability of their use in patients, practitioners and society, over time. What demands do living organisms make on patients and practitioners in contrast with chemotherapeutic, technological and non-living medical interventions? How are these differences managed? And how might our ways of thinking about and practising medicine have to change in response to a more than human perspective?

These and other questions reveal the importance of subjective and affective relationships in the medical encounter. Why do we feel obligations, take comfort in, or feel disgust toward, living therapies in ways that are diminished or absent with non-living medical interventions? How do we relate differently to a maggot than we do to a dog or a drug prior to the medical encounter? How does this change during and after? What makes possible, and what are the limits to, multispecies companion relationships? These questions are important as they open up to study the processes by which nature and culture are mutually constituted through medicine over time. They demonstrate the critical insight that the medical humanities can bring to understanding health as a product of more than human interactions.

Investigating why non-human life has been used to promote human health charts the social, political and economic contexts that facilitate the development and acceptance of medical interventions, including changing cultural perceptions of species difference. In recent years, examples of what we are calling ‘multispecies medicine’, including leech, larval and phage therapy, have found gained renewed medical interest having been pushed to the margins of 'scientific' medicine during the mid twentieth century. Others are applied in new ways, such as the development of deaf and disability assistance animals, as well as diabetes and seizure alert dogs, from the model of the guide (or ‘seeing eye’) dog. These examples powerfully impact on people’s lives not only by providing new therapeutic interventions but by producing new forms of multispecies companionship capable of transforming lived experience. Yet they are little studied leaving unanswered important questions as to how cultural and social values sustain and challenge human–animal relationships medicated by health. Why are some species over others acceptable as emotional therapy animals? Why are some social spaces resistant to the new forms of human–animal companionship on which therapeutic relationships are founded? How do therapeutic practices become positioned as integral, alternative and complementary to medicine? Answers to these questions lie in cultural and social values. As such, they are questions for the medical humanities. Understanding what these positions mean, as well as how and why specific therapies move across these categories over time, is critical to understanding how cultural perceptions shape medicine. And equally critical to understanding the everyday experiences of those whose health and well-being are shaped by their non-human medical companions.

Investigating the consequences of utilising non-human life to promote human health and well-being reveals how medicine contributed to, while being transformed by, the late 20th century emergence of categories such as ‘companion animal’ and the ‘human–animal bond’. These latter concepts are conventionally associated with animal advocacy politics, often used to critique laboratory experimentation on animals. From our perspective, however, these concepts, now fundamental to animal advocacy politics, the scholarly study of human–animal relations, and some forms of post-humanism, historically co-developed with and within medicine. It was, for instance, 20th century veterinarians, physicians and psychiatrists, in seeking to establish that certain non-human animals could enhance human health and well-being, that new categories of animal such as the emotional support animal could come into being. The mobilisation of the human–animal bond in the service of health illustrates a shift in medical thinking. Concepts such as symbiosis and interdependence, historically marginal to Western scientific medicine, are recovered and deployed as useful frames for understanding life, health and well-being in the early 21st century.12 Indeed, the resurgence of interest in processes of interdependence across the humanities, social and natural sciences contains the potential for synergistic interdisciplinary conversations.13 Arguably, the medical humanities are well positioned to facilitate such engagements. For the medical humanities the philosophical consequences are significant. Within the papers which make up this Research Forum, the fundamental category of the possessive individual and the valuing of independence as the measure of health give way to the category of collectives, the positive recognition of interdependence, and the extension of companion relations beyond the human.14 Such a move has direct social relevance. Exploring how ‘multispecies medicines’ contribute to, and draw from, wider sociocultural interest in ‘companion animals’ and the ‘human–animal bond’, together with the broader intellectual and political landscape of animal advocacy, reveals how health and well-being shape and are shaped by society. Charting how medicine productively contributed to reshaping human understanding of, and relations to, non-human animals, while being transformed in the process, will thereby enhance understandings of the relationships between medicine, animals and society.

A number of themes worthy of further investigation emerged from the 'Being well together' workshop, each explored further in subsequent articles that make up this inaugural Research Forum. One overarching theme was the ease with which non-human animals taking on a role within human health could be characterised as transgressive. Such practices challenge the cultural values and related expectations which sustain medical and societal structures. Using maggots in a clinical setting to aid wound healing, for instance, sits uncomfortably with the medical expectation of a hospital ward conforming to the highest standards of cleanliness and hygiene. Maggots themselves further challenge foundationalmedical categories. As neither a pharmaceutical drug nor a device, these living medical companions fit uneasily within existing regulatory frameworks. Practically, their care and use poses challenges to the conventional organisation of modern healthcare. Guide and other assistance animals raise similar tensions; are they to be understood as mechanical tools or as living organisms? Beneath attempts to render animals as tools, devices and machines, one finds a deeply organic relationship where shared affect and companionship is central to achieving the partnership’s ends. This dynamic is examined by Neil Pemberton’s historical study of the guide dog. Whether the animal is viewed as a technology or a living organism is shown to have real consequence. The practical success of the ‘human–dog’ partnership relies as much on affective intimacy as it does on standardised training. The ontological framing of the animal equally shapes representations of the lived experience of disability, of identity and the political consequence of framing disability as individual condition or an outcome of the physical and social environment.

The medicalisation of human–animal relationships increasingly challenge societal expectations of where and when the presence of animals is acceptable in public spaces. Justyna Włodarczyk, for instance, in her study of emotional support animals, reveals how the perception of some spaces as ‘human’ (such as airports and aeroplanes) enables discursive critique and dismissal of mental health needs through mechanisms that would otherwise be recognised as deeply irresponsible. In contrast, Rich Goman (in a forthcoming Research Forum) charts how the repurposing of livestock farms and animal sanctuaries to operate as therapeutic spaces has been received relatively positively (although not unproblematically from a critical animal studies perspective). Gorman’s ethnographic analysis explicitly argues that spaces of care farming can be ‘mutually therapeutic’– positive experiences for human and animal alike.

Many more than human therapeutic practices evolve from grass roots and often patient led initiatives. In a forthcoming paper, Alice Beck outlines how the ‘hacker’ ethos supported by internet communities has facilitated the rapid contemporary emergence of faecal matter transplant. One reason for sidelining practices of ‘multispecies medicine’ might, therefore, be their location outside the conventional structures of professionalised healthcare. Equally, they sit at the hinterland of health and social need. Animals often serve to support complex entanglement of health and social needs. The guide dog, for instance, allows the visually challenged to enter into society, navigate the world with renewed confidence, and as such provides life changing experiences which impact on health and well-being through reintegrating an individual into a (more than human) social community. Similar roles, as a future paper by Helen Brookes, Kelly Rushton and colleagues will illustrate, are played by common pet or companion animals in sustaining social and emotional well-being.

A provocative paper by Marie Fox and Mo Ray reveals the significant challenge that entering a care home can pose to the human–animal relationship. Nursing homes routinely separate human from animal disregarding the impacts this may have which has been likened to a form of grief. Here we again witness the inseparable character of social and health needs, starkly revealed through a shared vulnerability. For Fox and Ray, the integral contribution that animals make to human mental, emotional and social well-being invites the question as to whether the legal definition of family should not be widened beyond the human to include companion animals.15 The point of separation at the care home renders human and animal newly and radically vulnerable. Whether such a phenomena can, or should, be studied equitably is an open question as the way in which species difference forces unequal power relations is both impossible to ignore yet difficult to theoretically grapple with. Within the medical world, this question links to ethical concerns as to whether the use of animals to promote human health should be seen as beneficial to or an exploitation of the non-human. Within the 'Being well together' workshop and the papers which make up this Research Forum, answers were neither in the affirmative or negative. Rather, contributors describe varied and messy situated constellation that incorporate elements of both. Although, operating at apparently different ethical registers which become ever more visible at two diametrically different scales. At one end are cases involving the larger mammals, such as dogs and horses used as assistance animals, what one participant labelled the 'grand' therapeutic animals. At the other were invisible fellow travellers, companion creatures at the microbial level who live within us and on us.

## The ‘grand’ therapeutic animals and disability studies

If human relations to non-human animals have consequence for health and well-being then the medical humanities becomes a distinctive space within which new coalitions can be forged. Not least between disability and animal studies. Productively aligning animal and disability studies promises new possibilities for thinking through questions that are of paramount significance to the medical humanities, questions concerning shared health, vulnerably, interdependencies and social justice. Such a move, however, has to be approached with care. Disability and animal studies have a tense relationship as scholars in both fields recognise the challenges to be overcome to build shared intellectual ground. A significant obstacle is a historically inherited discourse wherein people with disabilities have been excluded from the category of human and ‘animalised’ as a means to segregate, disempower and ultimately police the place of disability is society.16 Consequently, attempts to destabilise the human (the raison d'être of animal studies) risk being perceived as a threat to those who seek therefore to secure for themselves such status. Disability activists have successfully argued for inclusion and secured civil rights and liberties that are now widely protected by anti-discriminatory legislation. However, as a consequence, disability identity is deeply invested in ‘human’ rights. Given this history, and the high stakes involved, attempts to critically deconstruct the human category can at best be viewed with caution. For disability studies, the boundary between human and animal is a central tenet that enabled a critique and thus escape from the notion that ‘to have a disability is to be an animal’.17

On the other hand, it was the very existence of such a boundary that made historical practices of dehumanisation and disempowerment of the disabled possible. Accordingly, some scholars see political value in the recognition of shared vulnerabilities across species. The work of Sunaura Taylor, for example, is committed to aligning disability and animal scholarship seeking social justice for both. Taylor’s work asserts that the comparison of disabled to animal operates to disempower only through the unexamined acceptance of ‘discrimination against non-human animals themselves’.18 In contrast, Taylor argues that disability scholarship must refuse to replicate the repudiation of the animal in order to defend the dignity of disabled people. She challenges her readers to ask how ‘those of us who have been negatively compared with non-human animals assert our value as human beings without either implying human superiority or denying our own animality?’19 For Taylor, there needs to be a critical revaluation of the cultural values and power dynamics that give these associations meaning. Moreover, she calls for a revaluation of the value of dependence, particularly the long established association of dependence and being a burden. It is this underlying logic which Taylor holds to account as the driver of discrimination; exploitation and abuse—of both human and non-human animals. How the medical humanities may serve as a space within which to operationalise such a proposition is explored further in Neil Pemberton’s contribution to this Research Forum.

Recognising the value and reality of interdependence is a prominent theme within animal studies and a feasible bridge to build alliances with disability studies. When extended to include health and well-being, it resonates well too with the inclusive agenda of the medical humanities to empower among other marginalised groups the ‘patient’. The avowedly post-humanist scholar Cary Wolfe has argued that animal and disability studies can together challenge the limitations of humanism, which for him idealises autonomy, normativity and completeness. As important as it is to extend ethical and legal enfranchisement to those previously excluded from it, Wolfe argues that there are dangers of what he calls the ‘fetishisation of agency’ in disability studies. 20 Being that it roots disability identity in the same binaries of independence/dependence, self/other that operate to devalue persons with disability. The pursuit of individual rights presupposes autonomous independent subjects contained within healthy individual bodies. It leaves no space to consider the value of life which is situated within relationships of mutuality and interdependence. It is in this space the approaches from animal studies may find new and provocative applications within the medical humanities.

Forging connections between disability studies, animal studies and the post-human, contends Wolfe, opens ‘new lines of empathy, affinity and respect for different forms of life, both human and non-human animal’ which are otherwise excluded from the humanisms privileging of a singular, autonomous ‘human’ subject. 21 By charting how the recognition of shared embodied vulnerabilities across species reveals new lines of empathy, affinity and respect, papers presented at the 'Being well together' workshop underlined this point. Pemberton’s contribution to this Research Forum builds on Wolfe’s proposal that a blind person with a guide dog transgresses conventional humanistic categories. In contrast with the guide dog being presented as a mere object that ‘ables’ the blind person and integrates them within society, Pemberton’s detailed empirical study can be read as an example of what Wolfe characterised as:

an irreducibly different and unique form of subjectivity–neither Homo sapiens nor *Canis familiaris*, neither “disabled” nor “normal” but something else altogether, a shared trans-species being in the world constituted by complex relations of trust, respect, dependence and communication (anyone who has ever trained–or relied on—a service dog would be the first to tell you).22

Here, human–animal partnerships not only promote health and well-being but demonstrate other ways of being in the world, ways that go beyond the limits of humanistic categories.

From this perspective, the guide dog, as with other service animals, becomes much more than a utilitarian-esque tool or machine. Human and animal become united in intimate companionship. The resultant human–animal team act as manifest a cooperative and shared nature of being; health and welfare become an outcome of togetherness fostered through mutual regard, understanding care and attention.23 All too often the critical importance of these human–animal relations to those who live within and are part of them goes unacknowledged. Law, for example, frames guide and other service dogs as equipment, defining them in terms of the tasks they are trained to perform and how those jobs enhance the agency of individual human beings.24 Should the human partner enter a public space where the animal companion is unwelcome, such as a care home, the animal can and all too often is sacrificed with no recognition of the impact on the welfare of the human. Moments such as this exemplify how the health and well-being components of the human–animal relationship traverse the medical and social worlds.

## Imperceptible companions: considering the healers within

At the other end of the scale, 'Being well together' considered invertebrate and microbial non-humans which, increasingly, are being woven into the fabric of medicine. Not as entities to be eradicated but rather integral components of health and well-being. A prominent example is the US National Institutes of Health ‘human microbiome project’. Modelled on large scale biomedical endeavours such as the human genome project and the neuroscience focused BRAIN initiative, the human microbiome project is producing a genetic map not of the human body as conventionally understood but rather of its inhabitants: the trillions of microbes that live within, on and with us in everyday life.25 The microbiome evokes a vision of the body not multiple rather than individual. What makes the human, microbiomic accounts insist, is not so much a distinct set of organs or a particular genetic lineage, nor even a unique capacity for social behaviour and cooperation. Rather, it is a specific configuration of cellular and bacteriological life. What may be termed an ecological vision of health and well-being follows, as we begin to learn as humans we have never been individuals.26

From the perspective of the human microbiome project, we are first and foremost a multispecies ecology of microbial beings. 27 Some of the more provocative health implications of this claim featured at the 'Being well together' meeting with papers exploring ‘faecal microbiota transplant’ (FMT) therapy and the possibility that reputedly ‘parasitic’ hookworms might help alleviate autoimmune disorders. FMT is a fast emerging therapeutic practice within medicine that also has a significant ‘hacker’ do-it-yourself ethos driven by patient communities. In 2008, for instance, gerontologists at the University of Minnesota responded to persistent sickness caused by antibiotic resistant forms of *Clostridium difficile* by experimenting with transferring stools from patients with healthy guts to those experiencing infection. With a reported success rate of 94%, it initially seemed that FMT was destined to revolutionise the treatment of intestinal disorders. However, as previously alluded to, existing regulatory frameworks struggled to accommodate therapeutic interventions which incorporated living organisms. In 2013, the US Food and Drug Administration categorised FMT as a drug, requiring medical practitioners to embark on long bureaucratic processes to use the procedure for anything but *C difficile* cases.28 While the new dawn of microbiomic medicine would appear to be delayed in the wake of regulatory challenges, patients have taken it up themselves by establishing techniques and communities of exchange which have allowed FMT to flourish outside of healthcare regulatory frameworks. Participants share their gut microbiota with the aim of alleviating conditions for which ‘mainstream’ medicine appears unable to offer a treatment. Despite understandable fears surrounding the sharing of both tacit and formal microbiomic knowledge, the simplicity of FMT makes it very difficult to police. At the same time, FMT would appear to be circumventing established patterns of commercial biomedical development.

Microbiome therapies would seem to recast biomedicine within a radical ecological vision of health, evoking a ‘messy’ relational complexity within which medical humanities scholars should feel quite at home. Indeed, it contrasts vividly with some recent deployments of biomedicine as an explanatory framework within the humanities which appear to abandon the critical stance which is the hallmark of rigorous medical humanities scholarship (explicit for example in historians’ utilisation of archaeological DNA evidence or the wider ‘neuro-turn’ across the humanities). The concept of the microbiome, perhaps, heralds a different point of intersection between scientific and humanistic endeavour. Here the preservation of complexity (as opposed to its erasure), as well as the invitation to approach cultural and societal factors critically, promise common standpoints shared by the biomedical sciences and humanities alike. Here everything—up to an including the human—may be rendered open to critical reflection and revision. While we should be cautious of calls to establish ‘microbial humanities’ heralding ‘a whole new configuration of research… where arts and science are combined’ until we are confident the humanities and natural sciences are meeting as equal partners, there may well be commonground to be shared.

Such claims remain provisional. It is not immediately apparent that microbiomic approaches to medicine must herald a radical departure from existing biomedical paradigms. In evaluating the relevance of microbiomic research to that of the medical humanities, then, we would do well to heed Stefan Helmreich’s warning of a nascent ‘microbiomania’.29 The use of maggots and leeches in wound healing, for instance, operates not only through their macroscopically apparent actions (cleaning away decaying flesh, maintaining blood flow), but also in terms of the biochemical interactions associated with them (secreting digestive enzymes, injecting hirudin). One response to such more than human medicine has been to seek ways to replace such living organisms. Attempts are frequently made to anatomise their efficacy and reduce it to a suite of biochemical, synthesised and deployed within the pharmaceutical model. Or to replace the biological altogether with a mechanical device. 30 In a form of bio-prospecting, some researchers view these creatures as ‘living pharmacies'. The challenge being how to identify, extract and synthetically replicate their therapeutic biochemical. Contributors to this Research Forum, in contrast, chart an alternate path which embrace as opposed to erase the notion of health being an outcome of an entangled web of life. In this latter sense, microbiomic research complements multispecies ethnographic conceptions of life as always–already ‘multiple’ or ‘entangled’ and invites serious reflection on the grounds of such entanglement and its implications for the maintenance of healthy interspecies relationships.31

## Becoming well together and the Research Forum

We highlight here two radically different scales of ‘multispecies medicine’ not because they are different in kind, for the two are intimately entangled positing human–non-human relations as a site for promoting and maintaining health and well-being. Each case operates through a complex entanglement of cultural and biological factors framed within a societal context. Each varies in the degree to which it promises a radical reconfiguring of long established biomedical political tropes, or is open to incorporation within the same and a return of familiar individualistic politics of healthcare. Foregrounding the role of non-human forms of life in human health and well-being is a provocation to the medical humanities to consider what lies outside our current thinking. The language of ‘becoming with’ foregrounds our interdependencies, prompting recognition that what matters to an individual in large part must be placed into broader contexts of what matters to others. Within the medical humanities, this line of thought invites an approach to understanding health and well-being less as an essential property of an individual human and more as a shared outcome of relations between humans and non-human beings. In subsequent *BMJ Medical Humanities* issues, further articles will present case studies exploring how the experience of being well with animals reshapes understandings of medicine, health and well-being. We hope to show how the medical humanities can operate as a shared ground where the politics of animal and disability advocacy might be productively brought together under a shared programme. Forthcoming papers will address the lived experience of health as a product of multispecies relations, the role of affect and emotion in the maintenance of human and non-human well-being, and the societal politics of ‘being well’ when ‘being well’ is a more than human condition. Importantly, the Research Forum is not intended to serve as a monologue. In line with the underlying philosophy of multiplicity, relationality and shared becoming which motivates our intellectual interest in human–animal interactions as a site for the flourishing of health, the Research Forum is conceived as a shared space where readers and authors engage. We invite, therefore, critical responses from readers and look forward to the shared process of becoming together which will generate new perspectives on medicine, health and changing relations of human and animal life in society.

## Notes

1. Vinciane Despret, 2004. The Body We Care for: Figures of Anthropo-Zoo-Genesis. *Body & Society* 2004; 10: 111–34. Such claims rest on a revival of process philosophy, e.g. Isabelle Stengers (2008). A Constructivist Reading of Process and Reality. *Theory, Culture, and Society* 2008; 25:91–110. This literature is closely allied to themes and methodologies well established within the medical humanities, see Bruno Latour (2004), B How to Talk about the Body? The Normative Dimensions of Science Studies. *Body and Society* 2004; 10: 205–229.

2. D Haraway, 2008 *When Species Meet.* Minneapolis: University of Minnesota Press, 2008. Harway innovatively develops her ideas through the work of a number of writers, not least Karen Barad, Vinciane Despret and Isabelle Stengers.

3. For a recent overview see K Kalof (2017). *The Oxford Handbook of Animals Studies.* Oxford: Oxford University Press, 2017.

4. Held together by an interest in the study of interactions between human and animals, this field is alternatively known as animal studies, human animal studies, human-nonhuman-animal studies and anthrozoology. Much like debates within medical humanities (also known as health humanities) debates over name rest on nuanced issues related to identity, politics of inclusion, and relationships to wider stakeholders, practitioners and publics.

5. Franklin Ginn et al, 2014. Flourishing with Awkward Creatures: Togetherness, Vulnerability, Killing. *Environmental Humanities* 2014; 4:113–123.

6. Jo C Dumville et al, 2009. Larval therapy for leg ulcers (VenUS II): randomised controlled trial. BMJ 2009; 338: b773; C Durrant et al. (2006). Forgotten digital tourniquet: salvage of an ischaemic finger by application of medicinal leeches. *Ann R Coll Surg Engl* 2006; 88(5):462–464.

7. Beck Alan and Katcher Aaron, 1996 *Between Pets and People The importance of animal companionship.* West Lafayette, Indianna: Purdue University Press 1996.

8. Kirk R G W., Worboys, M. Medicine and Species: One Medicine, One History? In M. Jackson *The Oxford Handbook of the History of Medicine.* Oxford: Oxford University Press, 2011 pp.561-77.

9. P M Conn, 2008. *The Animal Research War.* Basingstoke: Palgrave Macmillan, 2008.

10. A Woods et al, 2018. *Animals and the Shaping of Modern Medicine One Health and its Histories.* Basingstoke: Palgrave Macmillan, 2018.

11. K Hodgson and M Darling, 2011. Zooeyia: An essential component of “One Health”. *Can Vet J*. 2011; 52(2)189-191.

12. J Sapp, 1994. *Evolution by Association A History of Symbiosis.* New York: Oxford UP, 1994.

13. Examples from the natural sciences include Scott F Gilbert et al. (2010). Symbiosis as a source of selectable epigenetic variation: taking the heat for the big guy. *Philos. Trans. R. Soc. Lond., B, Biol. Sci*. 2010 365(1540):671–8.

14. C Wolfe, 2010. *What is Posthumanism?* Minneapolis, MN: Minnesota University Press, 2010; B Braun and S J Whatmore (2010). *Political Matter Technoscience, Democracy and Public Life.* University of Minnesota Press, 2010.

15. The changing of place of animals in society and the question as to their place within human social structures is a prominent theme within animal studies contributing to contemporary redefinitions of the ‘family’; see for example Franklin Sarah and McKinnon Susan (2002) (eds.) *Relative Values: Reconfiguring Kinship Studies.* Durham, NC: Duke Univeristy Press, 2002.

16. For instance D C Baynton, 1993. Savages and Deaf-Mutes’: Evolutionary Theory and the Campaign Against Sign Language in the Nineteenth Century. In Van Cleve John Vickret (1993), ed, *Deaf History Unveiled: Interpretations from the New Scholarship.* Washington, D.C.: Gallaudet University Press, 1993 pp.92-112; M Schweik Susan (2009), *The Ugly Laws: Disability in Public* (New York: New York University Press, 2009).

17. L J Davis, 2013. Introduction: Normality, power and culture. In L. J. Davis *the Disability Reader.* London: Routledge, 2013, pp.1-13, p.8

18. S Taylor, 2011 ‘Beasts of Burden: Disability Studies and Animal Rights’, *Qui Parle: Critical Humanities and Social Sciences*, 19:2 (2011), 191–222.

19. S Taylor, 2017. *Beasts of Burden: Animal and Disability Liberation.* New York: London, The New Press, 2017.

20. C Wolfe, 2009. *What is Posthumanism.* Minneapolis: University of Minnesota Press, 2009.

21. C Wolfe, 2010, *What is Posthumanism?* University of Minnesota Press, 2010, 127–28.

22. C Wolfe, 2010, *What is Posthumanism*? University of Minnesota Press, 2010,141.

23. Andrew Stevenson, 2013 in his ethnographic reflections on the guide dog partnership notes how such subjectivities and their constitutive relations keep changing, are remade over and again in their daily navigation together as partners, defining each other and conjuring ‘worlds they move through’ Andrew Stevenson, 2013, ‘Dog Team Walking: Inter-Corporeal Identities, Blindness and Reciprocal Guiding’. *Disability and Society* 28, vol 8 (2013): 1162–1167.

24. Kelly Oliver, 2016 Service Dogs, Between Animal Studies and Disability Studies. *Philosophia* 2016; 6(2)2:241–258.

25. Natio and nal Institutes of Health Office of Strategic Coordination, (The Common Fund) (2006-), ‘Human Microbiome Project,’ https://commonfund.nih.gov/hmp accessed 28/09/2018.

26. Scott F Gilbert et al, 2012. A Symbiotic View of Life: We Have Never Been Individuals. *The Quarterly Review of Biology* 2012; 87:325–341.

27. E Yong, 2016 *Contain Multitudes: The Microbes Within us and a Grander View of Life.* London: Penguin, 2016; Jessica R Houf, 2017, ‘The Microbial Mother Meets the Independent Organ: Cultural Discourses of Reproductive Microbiomes,’ *Journal of Medical Humanities* (online publication 17 Aug. 2017), doi: https://doi.org/10.1007/s10912-017-9468-y

28. E Yong, 2016 *Contain Multitudes* pp.234–5. Some medical researchers sought in response to develop synthetic, standardised microbiomic interventions that would obviate the need for the transplantation of faecal matter. The move from living organism to synthetic or mechanical alternative is a common trope seen in larval therapy, leech therapy and elsewhere.

29. S Helmreich, 2016. *Sounding the Limits of Life: Essays in the Anthropology of Biology and Beyond.* Princeton and Oxford: Princeton University Press, 2016, p.65.

30. Kirk R G W, Pemberton, N. *Leech.* London: Reaktion, 2013, pp. 161-165.

31. E Kirksey, 2014 ed. *The Multispecies Salon.* Durham, NC: Duke University Press, 2014.
